# How COVID-19 Hijacks the Cytoskeleton: Therapeutic Implications

**DOI:** 10.3390/life12060814

**Published:** 2022-05-30

**Authors:** Maral Aminpour, Stuart Hameroff, Jack A. Tuszynski

**Affiliations:** 1 Department of Biomedical Engineering, University of Alberta, Edmonton, AB T6G 1Z2, Canada; aminpour@ualberta.ca; 2 Department of Anesthesiology, The University of Arizona Health Sciences Center, Tucson, AZ 85621, USA; hameroff@u.arizona.edu; 3 Department of Psychology, University of Arizona, Tucson, AZ 85621, USA; 4 Center for Consciousness Studies, The University of Arizona, Tucson, AZ 85621, USA; 5 Department of Physics, University of Alberta, Edmonton, AB T6G 1Z2, Canada; 6 Department of Mechanical and Aerospace Engineering (DIMEAS), Politecnico di Torino, 10129 Torino, Italy

**Keywords:** SARS-CoV-2, COVID-19, microtubules, MTOC, low-intensity ultrasound

## Abstract

The SARS-CoV-2 virus invades and replicates within host cells by “hijacking” biomolecular machinery, gaining control of the microtubule cytoskeleton. After attaching to membrane receptors and entering cells, the SARS-CoV-2 virus co-opts the dynamic intra-cellular cytoskeletal network of microtubules, actin, and the microtubule-organizing center, enabling three factors that lead to clinical pathology: (1) viral load due to intra-cellular trafficking, (2) cell-to-cell spread by filopodia, and (3) immune dysfunction, ranging from hyper-inflammatory cytokine storm to ineffective or absent response. These factors all depend directly on microtubules and the microtubule-organizing center, as do cell functions such as mitosis and immune cell movement. Here we consider how the SARS-CoV-2 virus may “hijack” cytoskeletal functions by docking inside the microtubule-organizing center’s centriole “barrels”, enabling certain interactions between the virus’s positively charged spike (“S”) proteins and negatively charged C-termini of the microtubules that the centriole comprises, somewhat like fingers on a keyboard. This points to the potential benefit of therapies aimed not directly at the virus but at the microtubules and microtubule-organizing center of the host cell on which the virus depends. These therapies could range from anti-microtubule drugs to low-intensity ultrasound (megahertz mechanical vibrations) externally applied to the vagus nerve at the neck and/or to the spleen (since both are involved in mediating inflammatory response). Given that ultrasound imaging machines suitable for vagal/splenic ultrasound are available for clinical trials in every hospital, we recommend an alternative therapeutic approach for COVID-19 based on addressing and normalizing the host cell microtubules and microtubule-organizing centers co-opted by the SARS-CoV-2 virus.

## 1. Introduction

The novel coronavirus, severe acute respiratory syndrome coronavirus 2 (SARS-CoV-2), is the causative agent of COVID-19, or coronavirus-19 disease, one of the most challenging pandemics of all time. Although vaccination is a proven strategy for encompassing coronavirus disease 2019 (COVID-19), the emergence of SARS-CoV-2 variants has led to waning protection against the virus. Significant efforts are underway to repurpose existing drugs, circumventing the lengthy wait time needed for testing de novo design drugs. Nevertheless, a specific treatment for COVID-19 has yet to be identified, and innovative strategies must be devised and implemented to address this challenge. In the ongoing battle against COVID-19, though, little attention has been given to the role of microtubules and of the microtubule-organizing center (MTOC) or to non-pharmacological therapeutic strategies targeting inflammatory and immunological operations that may be beneficial for decreasing COVID-19-induced complications and improving patient outcomes (33–35). In this paper, we recommend, as a complement to anti-microtubule drugs, alternative therapeutic approaches that treat COVID-19 by addressing host cell microtubules and MTOCs co-opted by the SARS-CoV-2 virus. In this section we provide background information concerning the MTOC and cytokine storm (Section 1.1) and the role of microtubules and the cytoskeleton in COVID-19 (Section 1.2) as well as the molecular architecture of the centriole and cartwheel and the relation of them with microtubules (Section 1.2).

### 1.1. “Brain” of the Cytoskeleton and Cytokine Storm

Although SARS-CoV-2 belongs to a family of infectious viruses that also includes SARS and Middle East Respiratory Syndrome (MERS), SARS and MERS never reached pandemic levels, and this raises questions as to why SARS-CoV-2 is so difficult to contain and treat. To some extent, all infectious viruses invade and “hijack” host cell machinery to proliferate and complete their life cycle, but SARS-CoV-2 seems to have extraordinary adaptive abilities in this regard. There have been several models [1] and hypotheses [2] about the life cycle of SARS-CoV-2 [3,4,5]. The early phases that are typically necessary in order for the coronavirus to gain cell entry—e.g., binding and viral entry via membrane fusion [6] or endocytosis, priming of spike protein, uncoating of RNA, RNA replication in double-walled vesicles (DMV), and transcription—are relatively well understood. The evidence on the basis of which to correctly or completely describe the late phases such as the assembly of the N-RNA complexes with M, E, and S proteins at the endoplasmic reticulum-Golgi intermediate compartment (ERGIC) toward several possible exit routes in small exocytic single-layer membrane vesicles (SMV), large virus-containing vesicle (LVCP) secretory pathways [7], or deacidified lysosomal vesicles [8] before plasma membrane exocytosis, however, is still incomplete. There are still uncertainties and unknowns concerning the process by which virions are formed and enveloped during exocytosis. Indeed, a wide field of research remains to be explored in clarifying the mechanisms underlying the particle formation, budding, transportation, and egress of SARS-CoV-2. Correspondingly, there are many more prospective pathways (models and hypotheses) that need to be considered and addressed across the life cycle of the virus with respect to its interactions with the cytoskeleton and microtubules as an essential component of egress [9,10,11].

**Figure 1:**
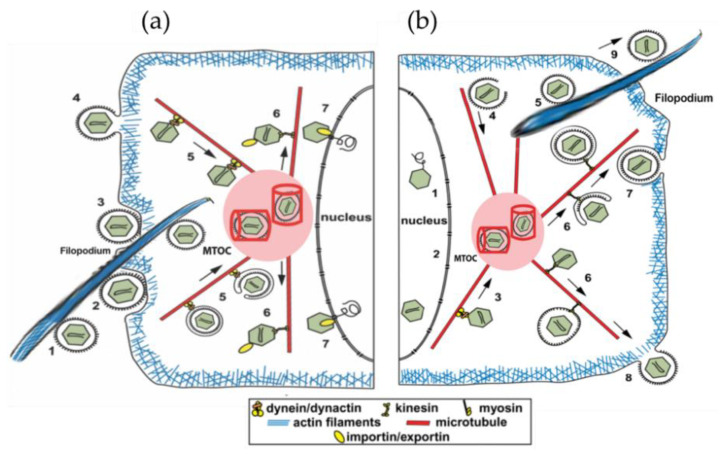
(**a**) Entry of SARS-CoV-2 into cells can take several routes: (1) virus delivered by filopodia extending from nearby infected cell; (2–4) fusion, invagination into cell; (5) transport by motor proteins toward MTOC; (6) transport from MTOC to different cell regions; and (7) entry into nucleus for reverse transcription and replication. The MTOC includes two perpendicular cylinders embedded in a dense electronegative “pericentriolar material”. The MTOC appears to enable optimal traffic along microtubules to distribute and replicate SARS-CoV-2 virus. (**b**) After reverse transcription and replication, virion is transported to MTOC (3,4,5) and from there along microtubules to other cellular regions and, ultimately, egress from the cell (6–9).

**Figure 2:**
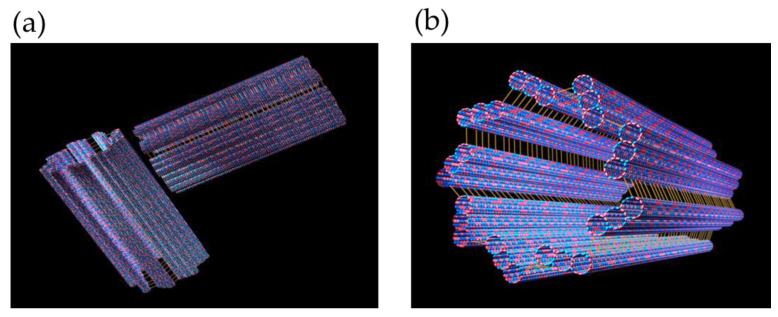
(**a**) A MTOC/centriole/centrosome comprising two cylinders, each consisting of nine longitudinally fused microtubules in a perpendicular array. (**b**) A single cylinder, i.e., half of a centriole/centrosome.

The particular cell machinery hijacked by SARS-CoV-2 and other viruses includes the actin and microtubule cytoskeleton. The cytoskeleton is a dynamic network of filamentous protein polymers, including microtubules, actin filaments, and centrioles, that functions as both the cell’s scaffolding and, apparently, an information processing and signaling system. The cytoskeleton forms part of the MTOC. Other microtubule-associated proteins (MAPs) connect cytoskeletal polymers into networks, and still others link them with membrane proteins. The focal point is the centrosome, or MTOC, which functions as the “brain” of the cytoskeleton, composed of a pair of microtubule-based cylinders, mutually perpendicular and embedded in an electronegative “pericentriolar material” (Figure 1 and Figure 2).

**Figure 3:**
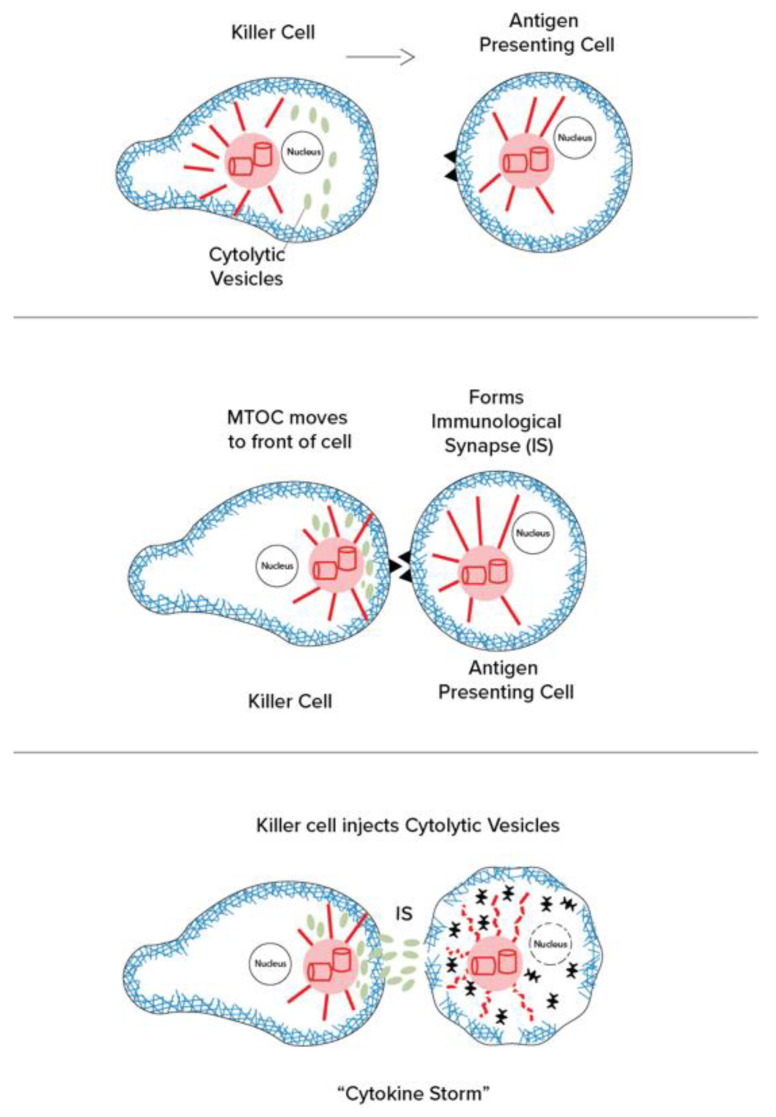
(**Left**): The effector cell (immune cell) makes contact and “immunological synapse” as the centrosome/MTOC moves to the “front” of the cell near the synapse. (**Right**): The centrosomes/MTOCs manage the release of cytolytic vesicles containing cytokines, tumor necrosing factor, interleukins, and other reactive agents (cytokine storm).

SARS-CoV-2 appears to use and depend upon co-opted activities of the microtubules and MTOC for infection, proliferation, and host cell damage. “Cytokine storm”, a hyper-inflammatory immune response causing extensive cellular damage, is organized and mediated through the MTOC (Figure 3).

### 1.2. Microtubules and the Cytoskeleton—Roles in COVID-19

The SARS-Cov-2 virus takes over human bodies by viral attachment, internalization, transport, transcription, replication, assembly, exocytosis, and cell-to-cell spread, causing further damage by inducing an excessive immune response on the part of the host, including “cytokine storm”, as well as by disrupting normal cytoskeletal functions. SARS-CoV-2 seems to be particularly adept at subverting and co-opting microtubules.

Microtubules, it should be noted, are cylindrical lattice polymers of the protein tubulin that are involved in critical cellular processes, including mitosis, cellular motility, and morphology, and the function of many cell membrane receptors. Trafficking of viruses and other material through cells is often facilitated by active transport by “motor proteins”, which convey cargo by efficiently moving it along these microtubules.

Microtubules self-assemble from tubulin, protein dimers composed of two similar 55 kDa monomer proteins, known as α-tubulin and β-tubulin, that self-assemble end-to-end to form long chains of tubulin dimers known as protofilaments. These then associate laterally into lattice sheets that roll into cylindrical microtubules. Adjacent protofilaments can associate in either of two ways corresponding to two types of lattices—the asymmetrical B-lattice with a seam and the seamless and hexagonal A-lattice with Fibonacci geometry and symmetry. Evidence indicates that microtubules have self-similar patterns of vibrational resonances and conductivity features at terahertz, gigahertz, megahertz, and kilohertz frequencies [12,13,14].

**Figure 4:**
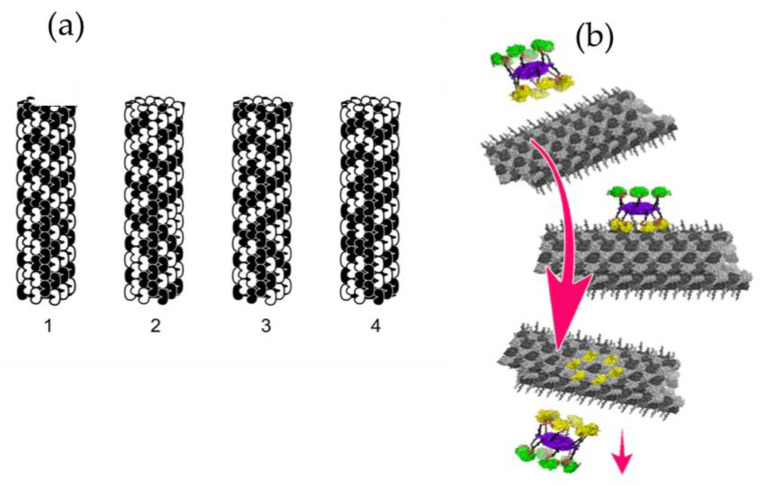
(**a**) Four steps in microtubule automata theoretical simulation. The state of each peanut-shaped tubulin protein is influenced by neighbor states in the microtubule lattice, resulting in moving patterns (1,2,3, and 4) capable of organizing cellular activities [16]. (**b**) In another theoretical simulation, a kinase (CaMKII) phosphorylates six tubulins in a microtubule lattice, encoding information. The hexagonal CaMKII precisely matches the microtubule A and B lattices [15].

Microtubules’ organizational abilities, lattice structure, and vibrational properties have prompted speculation that they process information, i.e., that they function as the cell’s nervous system and “on-board computer”. Each tubulin in a microtubule lattice can be modified (“encoded”) by post-translational modification and phosphorylated by kinase enzymes, making them ideal for information and vibrational microtubule regulation of cellular activities. It has been proposed in this regard that phosphorylation of specific tubulins in a microtubule lattice by synaptic CaMKII (calcium-calmodulin kinase 2) serves to encode memory in microtubules inside neurons (Figure 4) [15], and specific locations of the MAP tau on microtubules appear to regulate trafficking of motor proteins and their cargo. Other regulatory kinases may phosphorylate specific patterns of tubulins in microtubule lattices to initiate specific cytoskeletal activities, suggesting the occurrence of some manner of encoding.

Could SARS-CoV-2 hijack the cytoskeleton by hacking microtubule kinase codes, either by activating particular kinases or through direct effects on microtubules? If so, therapies aimed at disrupting microtubules may prevent virus spread and minimize damage to the host cell. Anti-microtubule drugs used in cancer chemotherapy to block mitosis, and in gout to immobilize immune cells, have already been shown to reduce viral load and to help in treating COVID-19 symptoms [17] and are currently being studied in human clinical trials [18,19].

The “centrosome”, or MTOC, contains two cylinders in a peculiar perpendicular array, each composed of nine parallel, interconnected triplets of microtubules, also known as the “centriole” (Figure 2). The MTOC centriole has been hypothesized, and to some degree demonstrated experimentally, to act as the cell’s cytoskeletal “brain”, and “eye”, capable of detecting electromagnetic signals in the visible and near-visible range [20]. Moreover, linked by microtubule spindles, the two cylinders replicate and separate to initiate cell division/mitosis and establish daughter cell shape. Single cylinders with nine parallel microtubule doublets and a central doublet also occur by themselves, providing the structure for cilia and flagella, which protrude, membrane-covered, from cell surfaces to serve sensing and cellular locomotion functions. Other cytoskeletal appendages include filopodia, composed mostly of actin, which are anchored, initiated, and sometimes occupied by microtubules. SARS-CoV-2 causes infected human cells to produce multiple filopodia laden with virus to extend outward toward neighboring cells, invade these cells, and spread the virus.

The MTOC appears to be the cytoskeletal command center, regulating cellular function and activities through microtubules and actin. Theoretical models [21,22,23] have suggested that microtubules process information, with individual tubulin subunit states representing information, and each tubulin dimer having two protruding, negatively charged C-termini “tails”. In this paper we consider how the SARS-CoV-2 virus may co-opt the cytoskeleton by docking inside centriole barrels of the MTOC, forming interactions between the virus’s positively charged spike (“S”) proteins and the negatively charged C-termini of the microtubules making up the centriole. In this manner, we suggest, the virus can modulate and interrupt MTOC and microtubule function, behaving in much the same way as fingers on a keyboard.

### 1.3. Molecular Architecture of Centriole and Cartwheel

**Figure 5:**
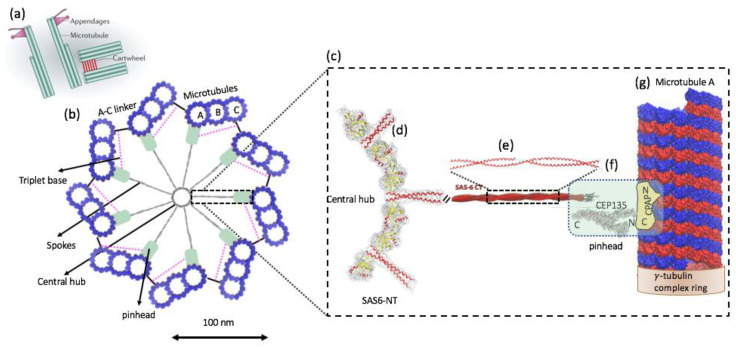
(**a**) Schematic representation of mother and daughter centriole pair in a human cell. (**b**) Schematic representation of the cartwheel and its associated proteins viewed from the proximal end (left). The cartwheel consists of a central hub (20~25 nm in diameter) from which nine spokes (40~45 long) emanate and connect through a pinhead (~20 nm) with A-microtubule of the peripheral microtubule triplets (blue). The A-microtubule from one triplet is connected with the C-microtubule of the next triplet located clockwise via a so-called A–C linker. The pinhead and the A–C linker are connected through the triplet base. The pink dashed line indicates the putative position of the triplet base. (**c**) A close-up view of the A-microtubule and its associated key proteins, such as hSAS-6, CEP135, and CPAP (right). A-microtubule is rotated 90 degrees from the view on the left. (**d**) The cartwheel’s central hub comprises SAS-6 homo-oligomers [26,30]. The central hub is not a continuous tube but rather a structure exhibiting periodicities along its 100 nm height. (**e**) The spokes extending outward from the hub are homodimeric SAS-6 coiled-coils (SAS-6 NT). (**f**) SAS-6 NT extends [30] into a region known as the “pinhead”, i.e., the celadon green color in the low magnification view (left) and in the rectangular box (right). CEP135 protein is located at the pinhead of the cartwheel and serves as a bridge between the spokes and the outer microtubules (possibly the A-tubule). It interacts with SAS-6 (via its C-terminal domain), microtubules s, and SAS-4 (via its N-terminus) [26]. CPAP carries both a tubulin dimer-binding domain [31] and a microtubule-binding domain [32] and is associated with the γ-tubulin complex [33]. (**g**) A-microtubule is a long, hollow cylinder made up of polymerized α- (blue) and β-tubulin (red) dimers. The outer diameter of a microtubule is approximately 25 nm, while the inner diameter is approximately 17 nm. The proximal end of the A-microtubule in a nascent human procentriole is capped by a structure similar to that of the γ-tubulin ring complex [34], a known microtubule nucleator in animal cells. ((**a**) [24] and part of (**e**) are adopted from [28] with author permission).

In human cells, the centriole is a cylindrical structure, typically 450 nm in length and with inner and outer diameters of ~130 nm and ~250 nm, respectively. Centrioles and procentrioles are characterized by a nine-fold radial symmetric arrangement of triplet microtubules that is polarized along its height, with the base referred to as the proximal end and the tip as the distal end. The proximal region of the centriole is defined by the presence of the cartwheel structure, which serves as an essential seed for centriole formation and is thought to participate in establishing the nine-fold symmetry of the entire organelle (Figure 5a) [24,25]. The cartwheel is built from a central hub (20~25 nm in diameter) from which nine spokes (40~45 long) emanate and connect through a pinhead (~20 nm) with A-microtubule of the peripheral microtubule triplets (Figure 5b). Each hub ring can accommodate nine homodimers of SAS-6, a protein that is essential for cartwheel assembly. The A-microtubule from one triplet is connected with the C-microtubule of the next triplet in a clockwise manner via a so-called A–C linker. The pinhead and the A–C linker, which are connected through the triplet base, extend more distal than the cartwheel and co-exist with the inner scaffold structure (the cartwheel is indirectly connected to the A–C linker through a flexible triplet-base structure extending from the pinhead). A close-up view of the A-microtubule and its associated key proteins on human centrioles and procentrioles, such as hSAS-6, CEP135, and CPAP, is shown in Figure 5c–g [26,27,28]. In this regard, a recent study applied cryo-electron tomography to four procentriole cartwheels of human cells (note that mature human centrioles lack cartwheels) in order to determine the cartwheel length and to better understand the relationship between the A–C linker and the cartwheel. This study found that the cartwheel length extends 189 +/− 9 nm and spans 70% of the length of the A–C linker (270 +/− 26 nm of the proximal region) in humans, where their study considered a procentriole length of ~410 nm [29].

## 2. Materials and Methods

The VMD molecular graphics software package is used for both the execution of Adaptive Poisson–Boltzmann Solver (APBS) [35] and the visualization of the resulting electrostatic potentials. AMBER force field [36] parameters such as atomic charges and radii are assigned using PDB2PQR webserver (http://server.poissonboltzmann.org, accessed on 29 May 2021) [37].

## 3. Results

### 3.1. Possible SARS-CoV 2 Invasion in Procentriole and Interaction with Microtubules

**Figure 6:**
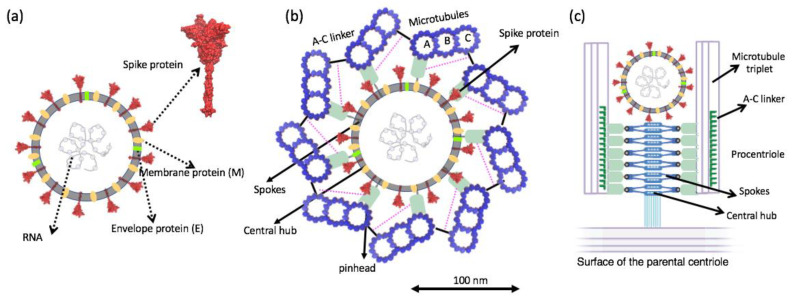
(**a**) Schematic of structural proteins of the SARS-CoV-2 virion. (**b**) Top view cartwheel complexed with the invading SARS-CoV-2 virion. Trimeric spike (S) protein on the surface of the SARS-CoV-2 is shown in red. (**c**) Side view of cartwheel-containing procentriole in complex with SARS-CoV 2 virion. (Part of (**c**) is adopted from [25] with author permission).

Electron micrographs of negative-stained SARS-CoV-2 particles show a generally spherical shape with some pleomorphism. The diameter of SARS-CoV-2, meanwhile, ranges between 50 nm and 140 nm [38]. As mentioned in the previous section, in human cells the centriole is a cylindrical structure, typically 450 nm in length and with inner and outer diameters of ~130 nm and ~250 nm, respectively. By comparing the size of the two structures, we can conclude that SARS-CoV-2 can physically fit within the centriole. In this study, we propose that the spike protein’s tip, which is seen to be relatively positively charged, docks onto the microtubule with the negatively charged C-terminus of the tubulin due to electrostatic attraction (Figure 6).

Regarding protein–protein interactions, many studies have examined the short-range interaction, which is dependent on the specific, complementary structure of each protein. However, the weak electrostatic binding interaction, which is probably effective over a long range, holds the same level of importance. A recent study showed that neutralizing the positively charged polybasic cleavage sites (R_682_RAR_685_ for each spike subunit), which are distributed approximately 10 nm away from the receptor-binding domain (RBD) of the spike protein, results in a weakening (by 34%) of the spike protein−ACE2 binding energy. It should be noted in this regard that ACE2 is highly negatively charged (−28 e), entailing that the extracellular membrane surface will be highly negatively charged. The dramatic drops observed in the RBD−ACE2 binding energy after neutralizing the polybasic sites with negatively charged mutations may be largely attributable to a change in (long-range) Coulomb interactions between ACE2 and the spike protein due to the RBD domain of the spike protein becoming more negative [39]. Microtubules are highly negatively charged due to the negative charge of the αβ-tubulin heterodimer itself (20–30 e−). However, a large portion of the tubulin charge, at least 40%, is concentrated in the non-structured C-terminal tails, which are rich in Glu and Asp amino acids.

**Figure 7:**
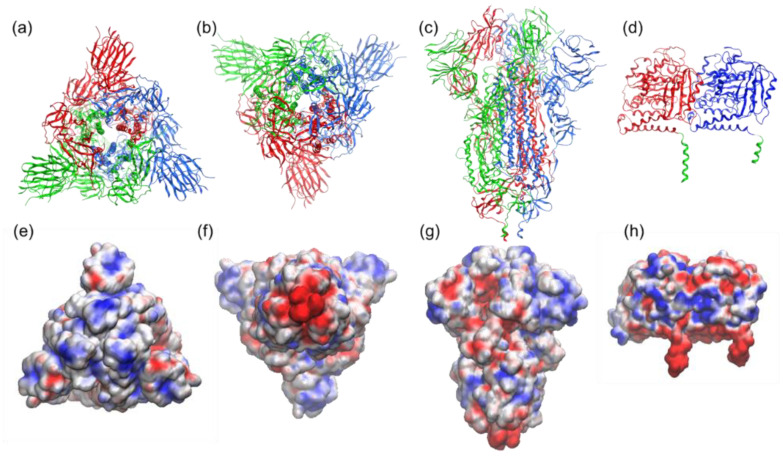
Structure and electrostatic map of the SARS-CoV-2 spike-S1 protein as viewed from (**a**,**e**) top (crown), (**b**,**f**) bottom (junction of S1 and HR2 linker; HR2 linker is not shown here), and, in (**c**,**g**), side, based on Protein Data Bank (PDB) entry 6VXX. The trimeric assembly of the spike protein (each monomer is colored in red, blue, and green) is presented as cartoon (first row) and as the electrostatic potential map (second row) highlighting a trefoil of positive charge (blue) around the central point. (**d**,**h**) Structure and electrostatic map of tubulin protein (PDB: 1jff) (α and β tubulin are in blue and red, respectively, and C-terminals are depicted in green). The VMD molecular graphics software package was used for both the execution of APBS (Adaptive Poisson–Boltzmann Solver) [35] and the visualization of the resulting electrostatic potentials. AMBER force field [36] parameters such as atomic charges and radii are assigned using PDB2PQR webserver (http://server.poissonboltzmann.org, accessed on 29 May 2021) [37].

**Figure 8:**
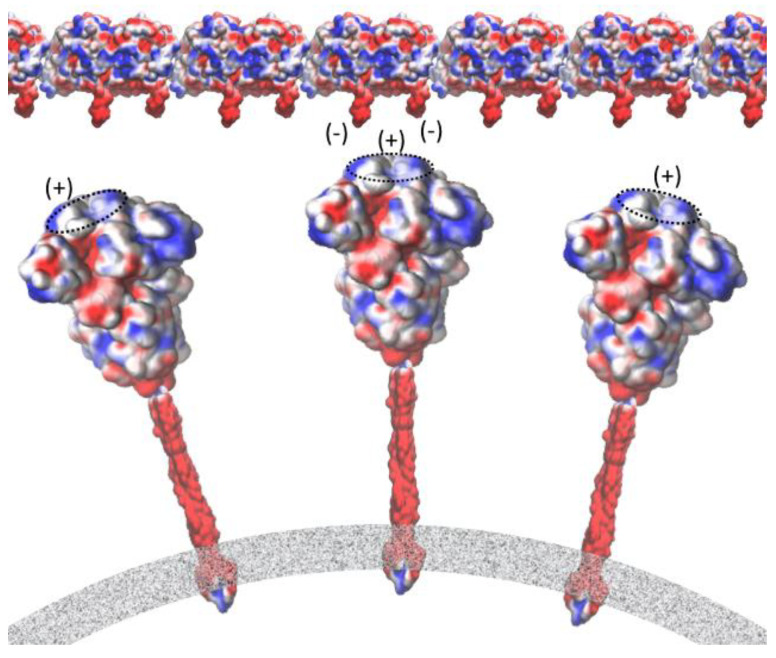
Interaction between the negative C-terminals of tubulin and the central point of the receptor-binding domain of the spike protein embedded in the membrane of the SARS-CoV-2 virion. Mapping of electrostatic potential (red = negative charge, blue = positive charge) was carried out using the VMD molecular graphics software package for both the execution of APBS [35] and the visualization of the resulting electrostatic potentials. Force field [36] parameters such as atomic charges and radii were assigned using the PDB2PQR webserver (http://server.poissonboltzmann.org, accessed on 29 May 2021) [37].

Several studies have characterized the interactions of various microtubule-associated proteins and the role of the negatively charged C-terminal tubulin tails in binding positively charged domains of these proteins to the tubulin surface [40,41]. Electrostatic potential at the binding interface provides insights into the role of electrostatic in the binding. Detailed information about the spike protein’s electrostatic surface can be obtained via the APBS calculation and the visualization of the resulting electrostatic potentials (see Figure 7 and Figure 8).

### 3.2. Electrostatic Potential at the Binding Interface

We performed the APBS calculations and visualized the resulting electrostatic potentials for the SARS-CoV-2 Spike protein (Figure 7a–c) and tubulin protein (Figure 7d). The electrostatic potential map of the spike protein (red = negative charge, blue = positive charge) highlights a trefoil of positive charge (blue) around the central point of the bulbous head (i.e., the central area of the receptor-binding domain). The C-terminals (two tails) of α and β tubulin monomers, meanwhile, feature the negative charge. Figure 8 provides a schematic representation of the attraction between the negative C-terminals of tubulin profilament and the central point of the receptor-binding domain of the spike protein embedded in the membrane of the SARS-CoV-2 virion.

## 4. Discussion

### 4.1. Snatching and Hijacking Microtubules

While the virus may be difficult to kill or avoid, most infections are not serious if the following three factors are minimized: (1) excessive viral load due to host cell entry, intra-cellular transport, and replication; (2) cell-to-cell spread by filopodia; and (3) immune dysfunction, ranging from hyper-inflammatory cytokine storm to inadequate response [42]. These factors all depend on the microtubules and MTOC, underscoring the potential benefit of therapies aimed not at the virus, but at microtubules and MTOC of the host cell on which the virus depends. There are at least three types of microtubule-based activities hijacked by SARS-CoV-2, as briefly described in the following subsections.

#### 4.1.1. Entry and Transport Inside the Host Cell

SARS-CoV-2 attacks a host cell through the coronavirus spike (S) protein, which mediates host cell attachment and viral entry [43]. The S protein attaches to membrane receptor proteins, primarily the ACE2 receptor, which in turn is associated with β-tubulin and anchored by intra-cellular microtubules. The virus then invaginates with surrounding membrane into the cell (becoming a “virion”) and then attaches to a microtubule via a motor protein. The motor protein then moves along the microtubule to bring the virus to the MTOC near the nucleus at the cell center that redirects virions. From there, virions may enter the nucleus, where coronavirus RNA exits the virion for reverse transcription and replication to a DNA replicon. This is subsequently transcribed back into RNA, which exits the nucleus into the MTOC. From there it is transported by different motor proteins moving along microtubules to various cell locations, including back to the cell membrane for egress from the cell. Virions also travel to other locations inside host cells, transported to specific areas by motor proteins along multiple cytoskeletal structures in what can be described as an on-demand “ride-share” system for virion transport inside host cells. Viruses rearrange these cytoskeletal filaments so that they can either utilize them as tracks or move them aside when they represent barriers (Figure 1).

#### 4.1.2. MTOC (Microtubule Organizing Center) and Cytokine Storm

The centrosome, or MTOC, organizes and directs intra-cellular movement and activities. For example, in mitosis/cell division, centrosomes replicate and separate to pull apart duplicate sets of chromosomes by means of microtubule mitotic spindles to establish daughter cell genome and architecture. In motile cells such as fibroblasts, which move into wounds to enable healing, the centrosome/MTOC is located in the front end of the cell, directing the “leading edge” of the actin to move in the proper direction. In the immune cells that move about to find infectious agents and antigen-containing cells, the centrosome/MTOC is behind the nucleus, still playing a key role in directional guidance and motility. However, when an immune cell, such as a killer T cell, reaches an antigen-containing cell, it forms an “immunological synapse” (IS), and the centrosome/MTOC moves to the front, near the IS. From there, the centrosome/MTOC directs release of cytolytic substances, including cytokines, tumor necrosing factor, and interleukins, in a phenomenon referred to as “cytokine storm” (Figure 3).

#### 4.1.3. Invasive Filopodia

Cells hijacked by SARS-CoV-2 grow arm-like cytoskeletal extensions, or filopodia, which extend outward from the cell and carry the virus from cell to cell, enabling local spread. Filopodia are composed largely of actin but are initiated, steered, and occupied by direct interactions with microtubules. SARS-CoV-2 is thought to control microtubules by controlling or activating kinases—enzymes that regulate cell functions by a process of phosphorylation, depositing high-energy phosphate groups. However, SARS-CoV-2 may directly access microtubule information codes to regulate functions. Figure 1a depicts a filopodium from another cell carrying the virus to a host cell, while Figure 1b depicts a filopodium carrying a virus out of the cell.

### 4.2. Therapeutic Approaches

Since SARS-CoV-2 is highly dependent on microtubule activities, therapies aimed at microtubules may be efficacious in treating COVID-19, either alone or in combination with other interventions. These potential therapies include (1) anti-microtubule drugs (see Section 4.2.1), such as colchicine, taxol, and others (but with a much lower toxicity risk, e.g., noscapine), that impair the microtubule and MTOC function on which virus proliferation depends; (2) low-intensity ultrasound vagal stimulation at the neck surface to enlist anti-inflammatory vagal actions (see Section 4.2.2); and (3) low-intensity ultrasound to the spleen to modulate inflammatory actions of immune cells, possibly dampening the effects on MTOC-mediated cytokine storm (see Section 4.2.3), where the latter two approaches use low-intensity ultrasound to resonate with microtubules’ characteristic mechanical vibrations.

The cholinergic anti-inflammatory pathway (CAIP) has been proposed as a principal mechanism by which the brain, through the vagus nerve, modulates the immune system in the spleen [44]. According to this mechanism, in response to infection or injury, the parasympathetic vagus nerve transmits signals from the brain to the adrenergic splenic nerve, which consequently interacts with splenic immune cells. Stimulation of the vagus and spleen nerves triggers this neural-immune reflex and dampens the inflammatory response to infection or tissue injury [45,46]. Vagus nerve stimulation (VNS), it should be noted, refers to any technique that stimulates the vagus nerve (e.g., electrical or ultrasound impulses).

It is worth mentioning that neurons (also called nerve cells) are the fundamental units of the brain and nervous system, and they rely on microtubules for their structure and functions. Microtubules form bundles that are particularly prominent in neurons and in the nervous system. Non-invasive, painless ultrasound to modulate microtubules in the vagus nerve may be a preferable option as a therapeutic approach for patients infected with SARS-CoV-2 [47,48,49].

#### 4.2.1. Anti-Microtubule Drugs

Colchicine destabilizes microtubules by preventing free tubulin dimers from being incorporated into the microtubule structure and thus prevents the assembly/disassembly cycles which enable immune cell movement. Accordingly, colchicine is effective against gout, a painful inflammation of joint spaces due to the build-up of uric acid crystals. By disabling microtubules, colchicine blocks immune cells from entering and inflaming joints to fight the uric acid crystals, which are relatively harmless. In this case, the body’s immune response causes severe pain and swelling that is more problematic than any effect of the uric acid crystals themselves. A recent open-label, randomized clinical study of colchicine in COVD-19 patients showed significant improvement [50]. While colchicine is somewhat toxic, with renal and other effects, new, less toxic colchicine-like drugs are currently under development. Moreover, there are a number of anti-cancer drugs that affect microtubule stability, impairing mitosis to fight malignancy, that could also be efficacious against COVID-19. One such example is noscapine [51]. We also would like to mention that our study is indirectly corroborated by the efficacy shown in clinical trials of anti-microtubule drugs. Recently, COLCORONA (Colchicine Coronavirus SARS-CoV2), which is a microtubule-targeting compound that disrupts mitotic spindle poles in human cells, has been selected for the phase 3 trial to treat COVID-19 (https://clinicaltrials.gov/ct2/show/NCT04322682, accessed on 29 May 2021) [52].

#### 4.2.2. Vagal Modulation with Ultrasound

The vagus nerve, the longest nerve in the nervous system, innervates visceral organs and tissues throughout the body, mediating parasympathetic effects, boosting the immune system, and blocking inflammation. Electromagnetic stimulation of the vagus nerve at the neck reduces systemic inflammation and is being tried in COVID-19 patients [53].

Ultrasound consists of mechanical waves above audible range, i.e., from 20,000 Hz to hundreds of megahertz but primarily hundreds of thousands of kilohertz to the low megahertz range. This technology has been safely used for medical imaging for nearly 100 years. Moreover, high-intensity ultrasound has been used to cause lesions by heating, e.g., to ablate tumors, and mid-range intensities from the scalp have been used to open the blood–brain barrier to allow drugs to enter the brain. Low-intensity, sub-thermal intensities, meanwhile, have been used to cause healing of peripheral nerves, bone, and other tissues and, when delivered (focused or unfocused) to the brain, can cause mood elevation and physiological effects. Although the mechanism underlying the healing and mood enhancement effected by low-intensity ultrasound is uncertain, it may involve microtubules, known to have resonances in megahertz range. Moreover, the application of low-intensity ultrasound to chondrocyte and osteoblast cells has been shown to cause rearrangement of the cytoskeleton [54,55,56].

#### 4.2.3. Splenic Ultrasound

The spleen produces and stores blood cells, including immune cells, and also has significant vagal innervation. In an animal model of systemic inflammatory arthritis, ultrasound to the spleen showed significant resolution of arthritis in all joints [46]. In that particular study, the mechanism was attributed to vagal stimulation, but it could well involve modulation, rearrangement, and repurposing of microtubules and MTOC in immune cells that then promulgate throughout the body. In the case of COVID-19, splenic ultrasound may change cytoskeletal plasticity in immune cells, inhibiting the ability of the virus to control the MTOC and/or promote anti-inflammatory actions [57].

## 5. Conclusions

The damage to host cells that leads to clinical symptoms in COVID-19 is governed largely by three factors: (1) excessive virus load due to host cell entry, intra-cellular transport, and replication; (2) cell-to-cell spread by filopodia; and (3) immune dysfunction, ranging from hyper-inflammatory cytokine storm to inadequate response [42]. These factors all depend on the SARS-CoV-2 virus co-opting microtubules and MTOCs (also consisting of microtubules) for their own purposes, and this suggests possible benefits of therapies aimed not at the virus but at the microtubules and MTOC of the host cell on which the virus depends. In this regard, clinical trials for colchicine and other anti-microtubule drugs as pharmacological agents aimed at preventing and limiting COVID-19 infections are underway [50]. As a complement to these efforts, we propose clinical studies for the application of low-intensity ultrasound to the vagus nerve at the neck and to the spleen (either through the abdomen or posteriorly through the ribcage) as a therapeutic for COVID-19 patients.

In fact, vagal stimulation at the neck with electromagnetic devices is already being tested in COVID-19 patients [53], and vagal ultrasound has been shown to reduce inflammation in animals [58]. In humans, anecdotally, vagal ultrasound is sedating and safe, resulting in a slight decrease in heart rate, and is easy to apply at the neck. Ultrasound to the spleen, meanwhile, has been shown to improve inflammatory arthritis in animals, an effect attributed to vagal stimulation in the spleen [46].

Ultrasound consists of mechanical oscillations from 20 kHz to hundreds of megahertz, ~100 million per second. The mechanism underlying the physiological effects of ultrasound is not yet known, but it is presumed to involve microtubules, as they have resonant oscillations in frequencies ranging from tens of kilohertz to megahertz, gigahertz, and even terahertz. Centrioles/MTOC are known to be sensitive to terahertz and gigahertz, abruptly changing direction of cell movement in response to infrared light [20]. In cell cultures, ultrasound has been shown to cause microtubule-dependent neurite sprouting and optimal functional rearrangement of microtubule configurations [59,60].

The main purpose and goal of this approach is for the ultrasound waves to resonate, modulate, and normalize microtubules and MTOC, which normally regulate immune and other cells but, in the case of viral infection, have been hijacked by the virus for its own purposes. Specific resonance frequencies and patterns of pulsation for microtubule have been identified that may be optimal [12,13,14], and nonspecific ultrasound frequencies (e.g., from 8 MHz to 500 kHz) transmitted to the brain through the scalp and skull have been shown to enhance mood in human volunteers [61,62]. As such, the application of nonspecific ultrasound frequencies to the vagus nerve and/or spleen is reasonable to consider as a therapeutic for COVID-19 patients.

Vagus nerve stimulation could be a promising adjunctive therapy for the treatment of COVID-19 patients. It can also be beneficial when the patient is not responsive to other kinds of therapies, such as anti-inflammatory drugs (including NSAIDs and glucocorticoids), or is suffering severe side effects as a result. The main advantages of low-intensity ultrasound are that it is safe, painless, non-invasive, relatively inexpensive, and readily available. Ultrasound machines are found in virtually every hospital emergency department and intensive care unit and are commonly used for imaging in sick patients. While we wait for effective antiviral drugs, and for those who do contract COVID-19, we propose a non-traditional approach that addresses and normalizes the hijacked microtubule and MTOC cytoskeleton.

## Data Availability

The datasets used and analyzed in the current study are available from the corresponding author upon request.
